# Evaluation of simulation-based ultrasound education using a bladder simulator for medical students in Japan: a prospective observational study

**DOI:** 10.1007/s10396-022-01269-5

**Published:** 2022-11-29

**Authors:** Hiroki Maita, Tadashi Kobayashi, Takashi Akimoto, Takahiro Hirano, Hiroshi Osawa, Hiroyuki Kato

**Affiliations:** 1 Development of Community Healthcare, Hirosaki University Graduate School of Medicine, 5 Zaifu-Cho, Hirosaki-Shi, Aomori, 036-8562 Japan; 2 Department of General Medicine, Hirosaki University School of Medicine and Hospital, 53 Hon-Cho, Hirosaki-Shi, Aomori, 036-8563 Japan; 3 General Medicine, Hirosaki University Graduate School of Medicine, 5 Zaifu-Cho, Hirosaki-Shi, Aomori, 036-8562 Japan

**Keywords:** Aging, Ultrasonography, Point-of-care systems, Medical students, Urinary bladder

## Abstract

**Purpose:**

This study aimed to investigate the usefulness of ultrasound education for medical students using a bladder simulator.

**Methods:**

This prospective observational study included volunteer fifth- and sixth-year medical students. An intravesical urine volume measurement simulator and a pocket-sized hand-held ultrasound device were used. The ultrasound education comprised pre-learning, briefing, simulation, debriefing, and learning summary. The simulation consisted of two tests: bladder simulator cube evaluation and scenario-based clinical application. A self-rated confidence score of 0–10 points along with reasons for the scores was recorded before and after the ultrasound education.

**Results:**

Twelve participants (median age, 23 years; female, 75%) met the inclusion criteria and were examined. Participants’ bladder simulator cube evaluation and scenario-based clinical application test results were good, and the educational difficulty level was appropriate. The mean confidence scores for main unit operation, probe control, image acquisition, image evaluation and clinical application before the ultrasound education were 1.0, 1.8, 1.3, 0.8 and 0.1 points, respectively. The mean confidence scores after the ultrasound education were 5.8, 5.9, 5.4, 5.5 and 5.1, respectively, with significant increases for all items (*p* < 0.01). The positive impression categories that affected confidence scores after ultrasound education were related to device operation, image acquisition, image evaluation, clinical application and learning.

**Conclusion:**

Ultrasound education using a bladder simulator increases confidence scores by imparting competencies related to device operation, image acquisition, image evaluation and clinical application, and it improves students’ learning impression. This is a useful method for introductory ultrasound education for medical students.

**Supplementary Information:**

The online version contains supplementary material available at 10.1007/s10396-022-01269-5.

## Introduction

In Japan, an aging population and shorter hospital stays have led to diversification of healthcare delivery systems, including increased home-visit medical care [1] and nursing homes for older patients [2]. Ultrasound devices are useful diagnostic tools for physicians and nurses in diverse healthcare settings. Thus, an ultrasound device is called a “second” stethoscope as it is less expensive, more compact, and more portable [3, 4].

However, understanding ultrasound procedures requires considerable time and effort [5]. Therefore, standardized ultrasound training in medical school is desirable for physicians to utilize ultrasound devices effectively in medical practice [6]. Simulation-based education is well established in U.S. and European medical schools [7]. However, Japanese medical schools are still developing simulation-based education [8], making it difficult to adequately master ultrasound techniques, even during the initial residency period [9].

In general, mastering ultrasound techniques requires acquiring images accurately, interpreting them anatomically, and performing hand–eye coordination [10]. These skills are challenging to grasp; therefore, ultrasound training courses for systematic learning have recently been provided in Japan, mainly for primary care physicians [11]. Nurses also have increasing opportunities to use ultrasound in evaluating heart failure [12] and management during patient transport [13]. Thus, educating nurses is essential [14]. Bladder ultrasound has been of interest in introductory ultrasound education for nurses in Japan because it is easy to grasp and clinically useful [4, 15]. Ultrasound education for medical students often begins with a focused assessment with sonography for trauma internationally [16, 17]; however, we focused on the bladder, which has a simpler structure. Moreover, there are no reports evaluating the usefulness of bladder ultrasound education for medical students.

This observational study aimed to evaluate the effectiveness of a bladder simulator in introductory ultrasound education for medical students with limited ultrasound experience.

## Materials and methods

This observational study was conducted at Hirosaki University School of Medicine, and the protocol was approved by the ethics committee of Hirosaki University (approval number 2021-135). All data were fully anonymized at collection, and written informed consent was obtained from all the participants.

### Study participants

Volunteer medical students in their 5th and 6th years of study at the Hirosaki University School of Medicine were included in the study between March 2022 and June 2022. Participants who met one of these criteria were excluded from the analysis: (1) qualified as nurses or clinical laboratory technicians; (2) provided incomplete answers to the questionnaire; (3) did not provide written consent.

### Overview of ultrasound education

The protocol for ultrasound education was: (1) pre-learning, (2) briefing (confirmation of learning objectives), (3) simulation (undertaking the task), (4) debriefing (reflection) and (5) summary of learning [18]. Regarding pre-learning, a physician used short movies to lecture the participants on operating ultrasound devices and probes, obtaining images, and evaluating images and their clinical application, following the content of a commercially available textbook [15].

**Fig. 1:**
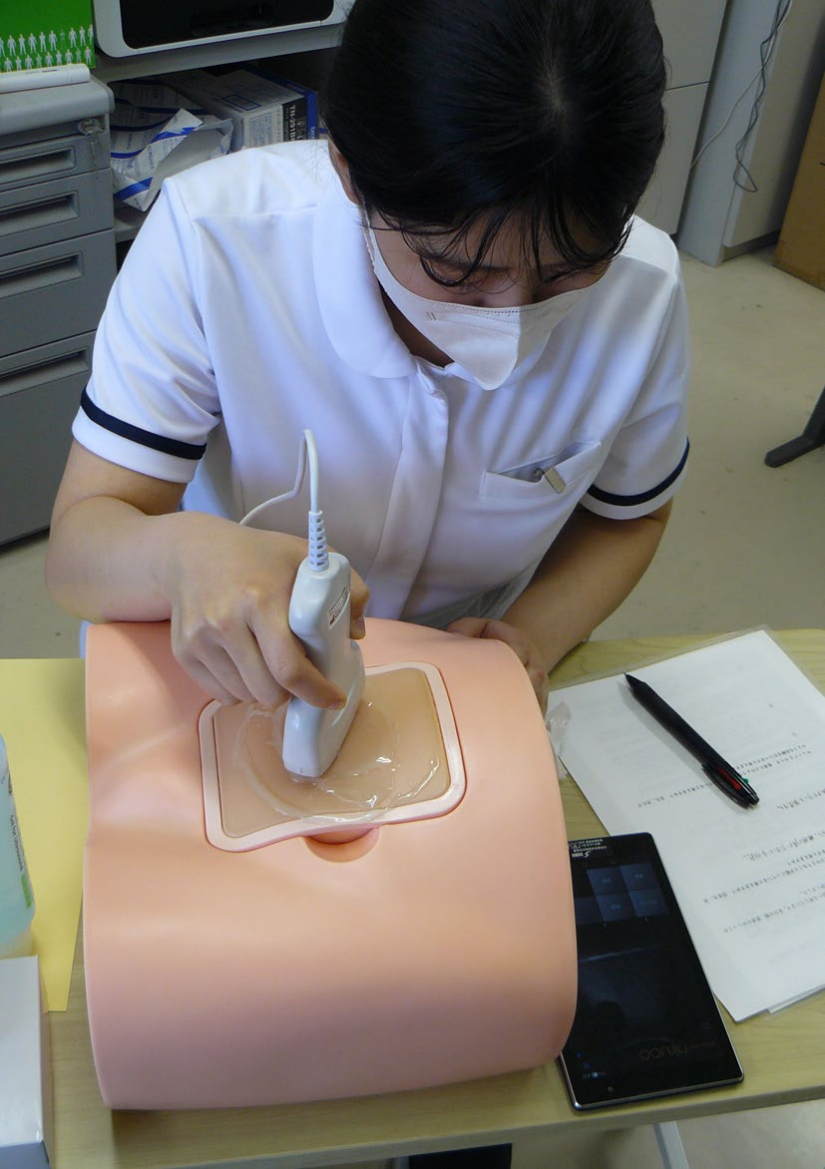
Student training with a bladder simulator and ultrasound device

The two objectives for the participants were: (1) to be able to estimate the bladder capacity and pathology and (2) choose a treatment method based on the ultrasound images and the patient’s medical history. The pre-learning lecture and execution of the tasks mentioned above were performed by a researcher, a family physician with over 20 years of clinical experience. First, participants were tested using a bladder simulator to distinguish between cubes of different bladder capacities (Fig. 1). The test was repeated until all four cubes could be correctly differentiated. Subsequently, clinical scenarios of urinary retention using a urinary retention with bladder catheter trouble cube and dehydration using a 50-ml cube were presented to the participants to test their clinical application skills. Finally, the participants made the diagnosis, prescribed the patient’s treatment in the presented scenario, and discussed the answer with the physician. For each scenario, a correct answer was defined as a clinically reasonable description of both diagnosis and treatment. Typically, the correct answers were intravenous infusion or drinking for dehydration and catheter replacement for urinary retention. Before and after the ultrasound education, a self-rated confidence score of 0–10 points was recorded regarding the competencies required to develop ultrasound skills (main unit operation, probe control, image acquisition, image evaluation and clinical application), and the reasons for the score were also recorded. Based on the above-mentioned process, individual feedback was given to the participants, and the learning was summarized.

### Ultrasound device

A pocket-sized hand-held ultrasound device (convex array probe, 3.5 MHz; body, miruco^Ⓡ^, Nippon Sigmax Co., Ltd., Tokyo, Japan) was used in this study.

### Simulator

An intravesical urine volume measurement simulator (Intravesical Urine Volume Measurement Phantom US-16, Kyoto Kagaku Co., Ltd., Japan) was used. This simulator can provide different ultrasound images by replacing four different cubes with different intravesical urine volumes (50 mL, 150 mL, 300 mL, and urinary retention with bladder catheter troublemodels).

### Data collection

Questionnaires were used to obtain information on medical school year, age, sex, ultrasound examination experience, the subject of ultrasound examination, the body part subjected to ultrasound examination, the institution where the ultrasound examination was performed and whether the participant had ultrasound training for clinical problem-solving. The number of times and time to answer all questions correctly were collected from the bladder simulator cube evaluation (Fig. S1). The number of correct answers was collected from the scenario-based clinical application (Fig. S2). Additionally, confidence scores before and after the simulator education and the reasons for the score were collected from the confidence score self-assessment form (Fig. S3). Confidence scores were rated on an 11-point Likert scale ranging from 0 (not confident at all) to 10 (confident that they could do it at the same level as doctors after completing initial residency).

### Qualitative and statistical analysis

Qualitative data, such as the reasons for the confidence scores, were coded and further categorized. The categories were classified into positive (feeling more confident, understanding, and gaining skills) and negative (feeling less confident, anxious, unsure, and insufficient skills) impressions. To compare Likert scales (confidence scores), a target sample size of 10 was calculated using an alpha error of 0.05, a beta error of 0.2, mean differences between the two groups estimated at 2, and a standard deviation of 2 to test the mean between the two groups [19]. NVivo version 1.6.1 and EZR version 1.54 were used for qualitative and quantitative data analyses, respectively.

## Results

Twelve volunteer medical students met the criteria, and their data (median age, 23 years; female, 75%) were analyzed (Table 1).

**Table 1:** Characteristics of the medical students (*n* = 12)

Age, median [years (IQR 25%, 75%)]	23 (22, 24)
Sex [*n* (%)]	
Female	9 (75)
Grade [*n* (%)]	
5th year	10 (83.3)
6th year	2 (16.7)
Number of ultrasound experiences prior to the study, median [*n* (IQR)]	2 (1.8, 4)
Subjects for whom the student performed ultrasound [*n* (%)]	
Patient	8 (66.7)
Simulator	6 (50)
Student	6 (50)
Supervising physician	3 (25)
Clinical laboratory technician	0 (0)
Nurse	0 (0)
Other	1 (8.3)
Body part where ultrasound was performed prior to the study [*n* (%)]	
Abdomen	7 (58.3)
Heart	5 (41.7)
Carotid artery	4 (33.3)
Uterus or ovary (transvaginal ultrasound)	4 (33.3)
Thyroid	2 (16.7)
Breast	1 (8.3)
Urinary bladder	1 (8.3)
Joint	1 (8.3)
Lung	0 (0)
Veins of the lower extremities	0 (0)
Other	2 (16.7)
Places where ultrasound was performed prior to the study [*n* (%)]	
University hospital	10 (83.3)
Non-university hospital	9 (75)
Clinic	0 (0)
Other	0 (0)
Clinical ultrasound training experience [*n* (%)]	0 (0)

*IQR* interquartile range

In the bladder simulator cube evaluation, the median number of attempts to correctly determine the bladder volumes of all four cubes was two times. The median time required to make a correct decision was 251 s. In the scenario-based clinical application test, 100 and 75% of the participants could provide accurate diagnosis and treatment in the urinary retention and dehydration scenarios, respectively (Table 2).

**Table 2:** Results of educational sessions using simulators (*n* = 12)

Bladder simulator cube evaluation	
Number of times to correctly evaluate all cubes, median [*n* (IQR 25%, 75%)]	2 (1, 2)
Time to correctly evaluate all cubes, median [second (IQR 25%, 75%)]	251 (172, 326)
Scenario-based clinical application	
Number of students who were able to make correct assessments based on clinical scenarios and ultrasound findings [*n* (%)]	
Scenario 1 (urinary retention)	12 (100)
Scenario 2 (dehydration)	9 (75)

*IQR* interquartile range

The mean confidence scores for main unit operation, probe control, image acquisition, image evaluation, and clinical application were 1.0, 1.8, 1.3, 0.8 and 0.1 points, respectively, before ultrasound education and 5.8, 5.9, 5.4, 5.5 and 5.1, respectively, after ultrasound education, with significant increases for all items (*p* < 0.01) (Table 3).

**Table 3:** Changes in medical students’ self-rated confidence scores before and after simulator training (*n* = 12)

Required skills	Mean confidence score, points (SD)		*P* value
	Before simulator training	After simulator training	
Main unit operation	1.0 (0.7)	5.8 (1.6)	< 0.01
Probe control	1.8 (1.0)	5.9 (1.4)	< 0.01
Image acquisition	1.3 (1.1)	5.4 (2.6)	< 0.01
Image evaluation	0.8 (1.0)	5.5 (2.2)	< 0.01
Clinical application	0.1 (0.3)	5.1 (2.2)	< 0.01

Paired *t* test was used to compare the mean confidence score

*SD* standard deviation

**Fig. 2:**
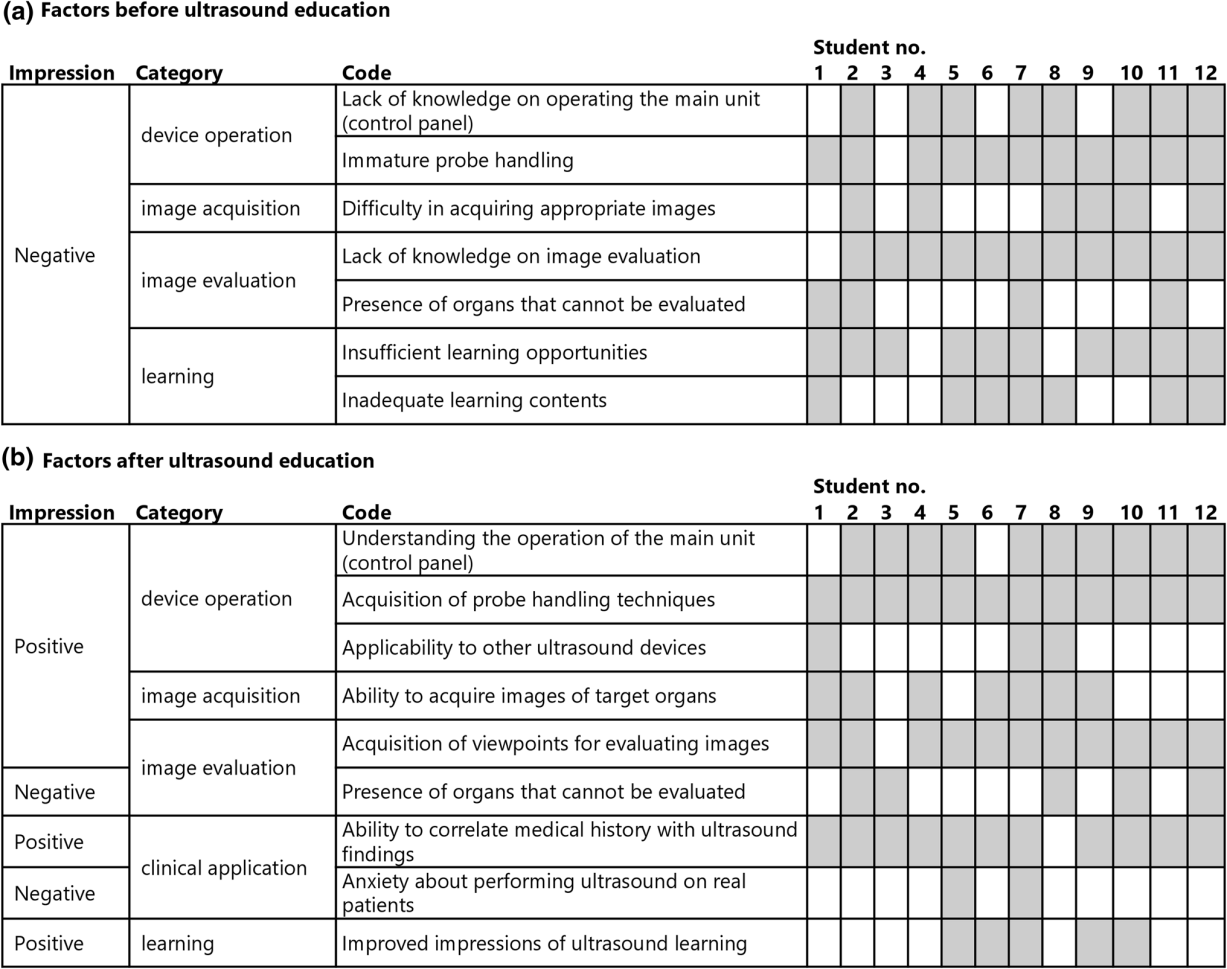
Factors (impressions, categories and codes) affecting confidence scores for ultrasound examinations before (**a**) and after (**b**) ultrasound education (*n* = 12). The gray cells in the figure indicate the existence of the factor

The categories that affected confidence scores before ultrasound education were device operation, image acquisition, image evaluation and learning, all negative factors for confidence scores. The categories that affected confidence scores after ultrasound education were device operation, image acquisition, image evaluation, clinical application and learning. The image evaluation and clinical application categories contained negative codes, including the presence of organs the participant could not evaluate (insufficient skills in assessing organs other than the bladder and prostate) or anxiety about performing an ultrasound on real patients. In contrast, other categories were positive factors for confidence score changes (Fig. 2).

## Discussion

This observational study included medical students with limited ultrasound experience and low confidence scores. The study revealed that ultrasound education using a bladder simulator improved participants’ confidence scores by imparting knowledge and skills in operating the ultrasound device (main unit and probe), acquiring images, evaluating images and clinical application, and it improved their learning impression.

In Japan, ultrasound education is increasingly being provided to medical students, clinical laboratory technicians, and nursing [20], pharmacy [21], physiotherapy [22], and acupuncture students [23]. However, from a global perspective, the quality of ultrasound education for medical students varies, and the implementation of systematic training programs has been delayed [24]. A survey of Japanese medical students reported that approximately 80% of the participants received abdominal or cardiac ultrasound training during their clinical training [25]. Our study participants also had a history of ultrasound training; nonetheless, they did not have sufficient opportunities for systematic and continuous training. As revealed in this study, the codes of insufficient learning opportunities and inadequate learning contents were negative factors that affected confidence scores before the simulator education. Due to inadequate training, comments such as “I ended up studying with a lack of confidence” were expressed. Thus, it is essential to provide learners with proficiency-based training rather than simply giving them learning opportunities. After the training conducted in the study, a code for an improved impression of ultrasound learning was extracted. Comments such as “There is no need to assume that ultrasound examination is difficult” were expressed, suggesting that training with a bladder simulator may maintain and facilitate learning motivation in students with limited ultrasound experience.

The three most common limitations in introducing ultrasound training into the medical school curriculum are a lack of trained instructors, availability of ultrasound equipment, and lack of educational opportunities within the tightly packed medical school curriculum [24]. As observed in a study of ultrasound education for nurses, the advantages of bladder ultrasound are the brief time required and the relative ease of performance [4]. Furthermore, the pocket-sized hand-held ultrasound device is inexpensive and portable. The participants’ opinion that “It was simple and easy to operate” indicates that the device is appropriate for beginners. The simplicity also allows the instructor to explain operating the device efficiently. For bladder ultrasound, it is easy to provide learning opportunities that meet the learner's schedule due to commercially available learning simulators. This study required a few minutes for participants to evaluate all four cubes (Table 2) correctly. The time required for pre-learning (consisting mainly of video clips) was approximately 10 min, which is not a big burden on the instructor if on-demand educational content and other resources are used. Therefore, we believe that using bladder simulators and pocket-sized hand-held ultrasound devices for introducing ultrasound education in medical schools is reasonable and effective in eliminating the learning barriers of instructor shortage, ultrasound device availability, and curriculum overload.

Simulation-based medical education uses simulations (such as manikins, virtual environments, and simulated patients) to educate students in clinical practice [26]. Simulation-based medical education includes task training to acquire individual skills and situation-based training to gain application skills through scenarios that simulate clinical situations [18]. This study applied a combination of task- and situation-based training. Generally, simulation-based education is based on a careful understanding of the learner’s readiness and goal setting. Specifically, education can be designed using the ADDIE model, on which many educational theories and simulation-based education are designed [27, 28]. The bladder simulator had an appropriate difficulty level with a reasonable increase in confidence scores (Table 3). Some participants noted that the simulator made it easier to compare images, which was an advantage over an actual patient. Codes of “improved impressions of ultrasound learning” were extracted after learning (Fig. 2), making the bladder simulator reasonably suitable for introductory ultrasound education. Negative codes (Fig. 2), such as the presence of organs that could not be evaluated and anxiety about performing an ultrasound on real patients, were also extracted after learning. We considered these negative codes as signs of changes in the learners’ needs and readiness (the ADDIE model’s analysis phase) to move to the next step of learning, given the comment “I don’t know how to evaluate other organs, but I will learn actively” for example. Therefore, developing a continuous curriculum that can be gradually applied to other ultrasound simulator education or actual patients, depending on the learner’s level, is required after bladder simulator education.

In Japan, as in other countries, the population is aging, and the number of older patients treated at home increases as their activities of daily living continue to decline [1]. Ultrasound would be utilized in countries with scarce medical resources and situations where the population is aging, and efficient use of medical resources is required. Particularly, pocket-sized ultrasound devices are highly portable and have fewer restrictions during use. The appropriate use of portable ultrasound devices in nursing homes and home care may assist in more appropriate decision-making for patients to visit specialized medical institutions at the right time, thereby reducing unnecessary medical visits [3, 29]. However, clinical residents are not confident enough to perform ultrasound, and acquiring proficiency in it requires considerable time. The American College of Emergency Physicians guidelines for point-of-care ultrasound suggest a minimum of 25–50 cases for each specific ultrasound area and subsequent cases to maintain the quality [5]. Therefore, effective and continuous ultrasound skill learning by medical, nursing, pharmacy and physiotherapy students is necessary. Introducing simulator-based ultrasound education, especially bladder simulators, for less experienced learners may contribute to solving the medical challenges of an aging society.

This study had several limitations. First, the study focused on the short-term evaluation of ultrasound education using a bladder simulator for medical students with limited ultrasound experience, which cannot be applied to long-term learning effects. Second, the study was based on a small number of medical students at a single institution, making it difficult to evaluate differences in student characteristics. Additionally, the appropriate time for introducing ultrasound education may differ among countries and educational systems. Third, the simulator education was provided by a single faculty physician, and its effectiveness may vary depending on the physician’s teaching style. Each educational session was conducted for a single student, which may have also resulted in more effective learning as the teaching could be adapted to each student's learning pace. However, results may differ with group education. Therefore, standardization is needed, as in ultrasound education for nurses [30]. Finally, the evaluation was conducted using a single portable ultrasound device and simulator, and evaluation with other devices is needed.

## Conclusion

Ultrasound education using a bladder simulator increases confidence scores by imparting competencies for device operation, image acquisition, image evaluation and clinical application, and it improves students’ learning impression. This method is useful for introductory ultrasound education for medical students with limited ultrasound experience.

## Supplementary Information

Below is the link to the electronic supplementary material.

Supplementary file1 (DOCX 16 KB)

Supplementary file2 (DOCX 17 KB)

Supplementary file3 (DOCX 17 KB)
