# Supplementary material for: Evaluation of simulation-based ultrasound education using a bladder simulator for medical students in Japan: a prospective observational study
Source: J Med Ultrason (2001). 2022 Nov 29;50(1):73–80. doi: 10.1007/s10396-022-01269-5 (PMC9892112; doi:10.1007/s10396-022-01269-5)
Supplement: Supplementary file 1 — Supplementary file1 (DOCX 16 KB) [file 10396_2022_1269_MOESM1_ESM.docx]

**Bladder simulator cube evaluation test**

**Question 1.**

You will be presented with a bladder simulator model with 4 different cubes (bladder capacity 50 mL, 150 mL, 300 mL, and urinary retention/catheter trouble model). Evaluate the bladder volume of each of the four cubes and check the boxes on the answer sheet.

**Answer Sheet**

**Cube 1.**

□ 50 mL □ 150 mL □ 300 mL □ Urinary retention (catheter trouble)

**Cube 2.**

□ 50 mL □ 150 mL □ 300 mL □ Urinary retention (catheter trouble)

**Cube3.**

□ 50 mL □ 150 mL □ 300 mL □ Urinary retention (catheter trouble)

**Cube 4.**

□ 50 mL □ 150 mL □ 300 mL □ Urinary retention (catheter trouble)
