# Supplementary material for: Evaluation of simulation-based ultrasound education using a bladder simulator for medical students in Japan: a prospective observational study
Source: J Med Ultrason (2001). 2022 Nov 29;50(1):73–80. doi: 10.1007/s10396-022-01269-5 (PMC9892112; doi:10.1007/s10396-022-01269-5)
Supplement: Supplementary file 2 — Supplementary file2 (DOCX 17 KB) [file 10396_2022_1269_MOESM2_ESM.docx]

**Scenario-based clinical application test**

**Question 2.**

You will be presented with a patient's medical history and a simulator model of the patient's bladder. You will examine the patient using an ultrasound device and describe on your answer sheet your decisions about (i) diagnosis of the patient and (ii) what treatment should be given next.

You will be presented with two different cubes in turn, and you should describe your decision for each cube.

**Answer Sheet**

**Patient's medical history**

80-year-old man with a chief complaint of inadequate urination visited your hospital. The patient is bedridden after a stroke and is staying in a nursing home. A staff member at the home noticed that the patient was not urinating this morning.

**Question 1.**

If the patient's bladder is in **cube 1**, (i) what do you think is happening to the patient (diagnosis)? (ii) Also, what treatment do you think should be given next?

(i)

(ii)

**Question 2.**

If the patient's bladder is in **cube 2**, (i) what do you think is happening to the patient (diagnosis)? (ii) Also, what treatment do you think should be given next?

(i)

(ii)
