# Supplementary material for: Evaluation of simulation-based ultrasound education using a bladder simulator for medical students in Japan: a prospective observational study
Source: J Med Ultrason (2001). 2022 Nov 29;50(1):73–80. doi: 10.1007/s10396-022-01269-5 (PMC9892112; doi:10.1007/s10396-022-01269-5)
Supplement: Supplementary file 3 — Supplementary file3 (DOCX 17 KB) [file 10396_2022_1269_MOESM3_ESM.docx]

**Self-rated confidence score form**

Please self-rate your current confidence for conducting ultrasound examinations on the following items on a scale of 0 to 10, (0, not confident at all; 10, able to perform at the same level as a physician after completion of primary residency). Also provide reasons for the score.

**Main unit operation**

0　　　1　　　2　　　3　　　4　　　5　　　6　　　7　　　8　　　9　　　10

(Reasons for this score)

**Probe control**

0　　　1　　　2　　　3　　　4　　　5　　　6　　　7　　　8　　　9　　　10

(Reasons for this score)

**Image acquisition**

0　　　1　　　2　　　3　　　4　　　5　　　6　　　7　　　8　　　9　　　10

(Reasons for this score)

**Image evaluation**

0　　　1　　　2　　　3　　　4　　　5　　　6　　　7　　　8　　　9　　　10

(Reasons for this score)

**Clinical application**

0　　　1　　　2　　　3　　　4　　　5　　　6　　　7　　　8　　　9　　　10

(Reasons for this score)
